# Commencement of the Pennsylvania College of Dental Surgeons

**Published:** 1860-05

**Authors:** 


### COMMENCEMENT OF THE PENNSYLVANIA COLLEGE OF DENTAL SURGERY.

MESSRS. EDITORS :—Annexed I give you a brief report of the commencement exercises of the College here.

*The Pennsylvania College of Dental Surgery* held their annual commencement on Wednesday evening, February 29, 1860, at the Musical Fund Hall, Philadelphia, and which attracted a large and fashionable audience.

The valedictory was delivered by Prof. T. L. Buckingham and elicited considerable applause.

The Dr. alluding to the high and noble aims of the profession and the paramount importance of those who practice it, to properly appreciate its mission and usefulness, said:

"Let these convictions, then, of the true worth and importance of your profession be added to such as you may have previously stored in your mind. Engage in your calling, with the honest belief that it is not simply an apology for making an honest livelihood, but a calling essential to the comforts and well being of society. And having such views of it, be excited by a laudable ambition to excel in your avocation."

By a reference to a printed statement, I find the class numbered *fifty-one*, and the Graduates *twenty-one*, including *two* from Cuba, *one* from Germany, and the balance of the graduates hailing from twelve different States of the Union—chiefly Southern.

The Demonstrator's report (operative department,) shows that 308 Patients were treated, making a total of 4,055 operations performed during the session in his department alone, in which are 612 gold fillings consuming, as I have been informed, over three hundred dollars worth of gold foil.

In the mechanical department, the Demonstrator reports, "whole number of teeth mounted 1,579," which includes twenty-four whole sets, forty-six upper and four lower sets of teeth.

The large number of operations performed prove conclusively the advantages enjoyed by the class, but at the same time give countenance to the complaint of some few of the city dentists, that the College is interfering with their practice in performing operations, gratuitously, that would otherwise come into their hands; and I must say, judging from appearances, a large number of those offering themselves at the clinic, were able to pay for the services of the dentist, and such, I certainly think, should be sent away, especially when there are such an abundance of those who can not pay; and also in view of the fact that not one in ten who offer, can be attended to.

I am fully aware that it is difficult to discriminate in many cases, and unpleasant to come in contact with the preferences of the Students, yet it is an evil unfair alike to the poorer classes and young practitioners, and as such should be guarded against, and if possible, corrected.

I am sure the gentlemanly demonstrators will receive these remarks in the spirit they are made, and believe, that my only object in noticing the matter is the general good.

The exercises passed off very pleasantly, and to the apparent enjoyment of the numerous auditory.

From a programme of the commencement exercises of the

*Baltimore College of Dental Surgery*, which was held on Tuesday evening, February 28, 1860, I learn that the number of *Matriculants* were *seventy*, and the *Graduates thirty-nine.* This, I believe, is the largest dental class ever attending that or any other school. Yours,

PHILADELPHIA, March 13th, 1860. O. U. C.

